# Molecular evolution of dimeric α-amylase inhibitor genes in wild emmer wheat and its ecological association

**DOI:** 10.1186/1471-2148-8-91

**Published:** 2008-03-24

**Authors:** Ji-Rui Wang, Yu-Ming Wei, Xiang-Yu Long, Ze-Hong Yan, Eviatar Nevo, Bernard R Baum, You-Liang Zheng

**Affiliations:** 1 Triticeae Research Institute, Sichuan Agricultural University, Yaan, Sichuan 625014, China; 2 Institute of Evolution, University of Haifa, Mt. Carmel, Haifa 31905, Israel; 3 Agriculture and Agri-Food Canada, Eastern Cereal and Oilseed Research Centre, Ottawa, Ontario K1A 0C6, Canada; 4 Key Laboratory of Crop Genetic Resources and Improvement in Southwest China, Ministry of Education, Sichuan Agricultural University, Yaan, Sichuan 625014, China

## Abstract

**Background:**

α-Amylase inhibitors are attractive candidates for the control of seed weevils, as these insects are highly dependent on starch as an energy source. In this study, we aimed to reveal the structure and diversity of dimeric α-amylase inhibitor genes in wild emmer wheat from Israel and to elucidate the relationship between the emmer wheat genes and ecological factors using single nucleotide polymorphism (SNP) markers. Another objective of this study was to find out whether there were any correlations between SNPs in functional protein-coding genes and the environment.

**Results:**

The influence of ecological factors on the genetic structure of dimeric α-amylase inhibitor genes was evaluated by specific SNP markers. A total of 244 dimeric α-amylase inhibitor genes were obtained from 13 accessions in 10 populations. Seventy-five polymorphic positions and 74 haplotypes were defined by sequence analysis. Sixteen out of the 75 SNP markers were designed to detect SNP variations in wild emmer wheat accessions from different populations in Israel. The proportion of polymorphic loci *P *(5%), the expected heterozygosity *He*, and Shannon's information index in the 16 populations were 0.887, 0.404, and 0.589, respectively. The populations of wild emmer wheat showed great diversity in gene loci both between and within populations. Based on the SNP marker data, the genetic distance of pair-wise comparisons of the 16 populations displayed a sharp genetic differentiation over long geographic distances. The values of *P*, *He*, and Shannon's information index were negatively correlated with three climatic moisture factors, whereas the same values were positively correlated by Spearman rank correlation coefficients' analysis with some of the other ecological factors.

**Conclusion:**

The populations of wild emmer wheat showed a wide range of diversity in dimeric α-amylase inhibitors, both between and within populations. We suggested that SNP markers are useful for the estimation of genetic diversity of functional genes in wild emmer wheat. These results show significant correlations between SNPs in the α-amylase inhibitor genes and ecological factors affecting diversity. Ecological factors, singly or in combination, explained a significant proportion of the variations in the SNPs, and the SNPs could be classified into several categories as ecogeographical predictors. It was suggested that the SNPs in the α-amylase inhibitor genes have been subjected to natural selection, and ecological factors had an important evolutionary influence on gene differentiation at specific loci.

## Background

Wild emmer wheat, *Triticum dicoccoides*, the progenitor of bread and pasta wheats, presumably originated in and adaptively diversified from, northeastern Israel into the Near East Fertile Crescent [1]. In this center of diversity, wild emmer wheat harbors rich genetic diversity and resources [1]. Previous studies in *T. dicoccoides *and other cereals have shown significant nonrandom adaptive molecular genetic differentiation at single and multilocus structures in either protein-coding regions or randomly amplified polymorphic DNAs among micro-ecological environments [2,3]. It was also determined that wild emmer wheat is genetically variable and that the genetic differentiation of populations included regional and local patterns with sharp genetic differentiation over short distances [4]. Genetic polymorphisms of α- and β-amylase in wild emmer wheat have been characterized, and it was found that diversity of climatic and edaphic natural selection, rather than stochasticity or migration, was the major evolutionary force driving amylase differentiation [5].

The estimates of molecular diversity derived from PCR-based techniques such as amplified restriction fragment length polymorphism (AFLP), microsatellites (short sequence repeats or SSR), single nucleotide polymorphism (SNP), and sequence comparisons are several-fold higher than enzymatic diversity [6]. A substantial private and public effort has been undertaken to characterize SNPs tightly associated for genetic diversity. SNPs are identified in ESTs (expressed sequence tags), thus the polymorphisms could be directly used to map functional and expressed genes, rather than DNA sequences derived from conventional RAPD and AFLP techniques, which are typically not functional genes [7-9]. The majority of SNPs in coding regions (cSNPs) are single-base substitutions, which may or may not result in amino acid changes. Some cSNPs may alter a functionally important amino acid residue, and these are of interest for their potential links with phenotypes [10].

α-Amylase is a family of enzymes that hydrolyze α-D-(1,4)-glucan linkages and play an important role in the carbohydrate metabolism of many autotrophic and heterotrophic organisms [11]. Heterotrophic organisms use α-amylase primarily to digest starch in their food sources [12]. Several kinds of α-amylase and proteinase inhibitors in seeds and vegetative organs act to regulate the numbers of phytophagous insects [13-15]. α-Amylase inhibitors are attractive candidates for the control of seed weevils as these insects are highly dependent on starch as an energy source [16]. In cereal seeds, α-amylase inhibitor proteins with 120–130 amino acids, which include trypsin inhibitors, as well as α-amylase inhibitors, can be grouped into one large family on the basis of the homology between their amino acid sequences [17]. In this family, the dimeric α-amylase inhibitor has been well characterized. For weevil control, α-amylase inhibitors could be manipulated through plant genetic engineering. However, many insects have several α-amylases that differ in specificity, and successful utilization of a food source is dependent on the expression of a α-amylase for which there is no specific inhibitor [12]. The dimeric α-amylase inhibitor genes were located on chromosome 3BS and 3DS; there was no known evidence of a homoeologous locus or loci on chromosome 3AS of the polyploid wheats [18,19]. Therefore, the tetraploid wheats, which are lacking the D genome, have only the inhibitor genes on chromosome 3BS [19].

Evolutionary pressures of various kinds have often been hypothesized to cause active and rapid evolutionary changes. In a co-evolving system of plant-insect interactions, plants synthesize a variety of toxic proteinaceous and nonproteinaceous molecules for their protection against insects [20,21]. Proteinase inhibitors are therefore a potential model system in which to study basic evolutionary processes, such as functional diversification [22].

It is well established that multiple forms of proteins are active on exogenous or endogenous α-amylases in the wheat kernel, and proteinaceous dimeric α-amylase inhibitors could function against α-amylase from various origins [23]. It is known that the bulk of wheat albumins consist of a few amylase iso-inhibitor families that are very likely phylogenetically related and coded by a small number of parental genes [24]. The α-amylase inhibitors have long been proposed as possible important weapons against pests whose diets make them highly dependent on α-amylase activity. *In vitro *and *in vivo *trials using α-amylase inhibitors, including those made under field conditions, have now fully confirmed their potential for increasing yields by controlling insect populations [16].

Two conflicting views confront ecologists and evolutionary biologists on the degree of symmetry in interactions between plants and phytophagous insects [25]. The symmetrical view holds that insects and plants have strong effects on one another's evolutionary and ecological dynamics. The asymmetrical view acknowledges that plants have major effects on insects but claims that insects seldom impose significant effects on plants [25]. Plant defense mechanisms have been the subject of intense investigation [26]. The genome shaping events and processes occurring at dimeric α-amylase inhibitor gene loci from the B and S genomes of wheat and *Aegilops *section sitopsis, respectively, have been characterized. A Phylogenetic Median-Joining network of the haplotypes and a neighbor-joining tree analysis have indicated that the inhibitor gene sequences from common wheat and *T. dicoccoides *are closely related to those from *Ae. speltoides *[27]. However, little is known about their evolution under the influence of ecology. The molecular diversity of α-amylase inhibitor genes, as well as their divergence among 16 populations of wild emmer wheat from Israel, was investigated to gain insight into the correlation between plant defense proteinaceous inhibitors and ecological factors.

## Results

### Isolation of the ORF of dimeric α-amylase inhibitors

Using two cloning primers, genomic PCR amplifications were conducted, and one desired DNA band was detected in each accession of wild emmer wheat. Cloning the fragments yielded 244 positive clones from 13 accessions (randomly selected from 10 populations), which were subsequently sequenced (data not shown). Only three out of 244 dimeric α-amylase inhibitor genes had a common three bp deletion, and those three genes were obtained from one accession derived from Mt. Hermon, whereas the other cloned fragments had 426 bp long (data not shown). It was predicted that all of the 426-bp sequences would encode functional dimeric α-amylase inhibitors. Alignment of the gene sequences from emmer wheat with sequences from the species of *Aegilops *section Sitopsis (including *Ae. speltoides*, *Ae. bicornis*, *Ae. longissima*, *Ae. searsii*, and *Ae. sharonensis*), *Ae. tauschii*, einkorn wheats, and common wheat clearly indicated that the emmer wheat sequences were derived from the B genome [27].

### SNP and haplotype analyses of dimeric α-amylase inhibitor genes

The frequency of SNPs in the dimeric α-amylase inhibitor genes in emmer wheat was 1 out of 5.7 bases, which was higher than the SNPs observed for kunitz-type α-amylase inhibitor and α-amylase/subtilisin inhibitor genes in barley and dimeric α-amylase inhibitor genes in common wheat [28-30]. Among the 426 nucleotides, there were 351 conserved positions and 75 variable positions among the 244 α-amylase inhibitor genes sequenced from 13 accessions.

**Figure 1:**
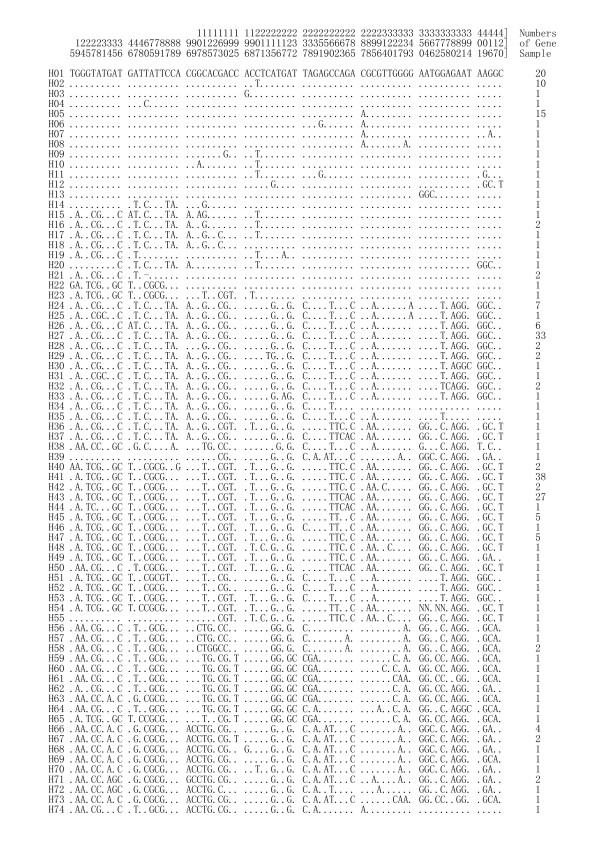
Haplotypes of 244 dimeric α-amylase inhibitors obtained from 13 accessions (10 populations) in Israel.

A total of 74 haplotypes were revealed by sequence analysis (Figure 1); 53 of these were each found in only a single sequence. Haplotype 41 was observed at the highest frequency, i.e., in 38 gene sequences, followed by haplotype 27 in 33 sequences (Figure 1).

The relationship between SNPs and amino acid changes in the α-amylase inhibitor proteins is summarized in Table 1. The 75 SNPs resulted in 38 amino acid substitutions. The position of each SNP in the sequence, whether the predicted change was synonymous (silent) or non-synonymous (replacement), was determined. Forty percent of SNPs were found to occur at the third codon position, and as expected, most of these were synonymous (Table 1). A number of changes were also identified in codon positions 1 and 2, and these accounted for more than 95% of the non-synonymous changes (Table 1). In total, 60% of the SNPs resulted in non-synonymous changes.

**Table 1:** The variation of amino acids caused by the nucleotide changes in genes. The SNPs were detected by primers at the position with bold numbers in the Site column.

Number	Site	Substitution	Amino acid position	Amino acid variation	Numbers of gene sample
1	5	T-G-A	Signal peptide	Leu-Arg-His	241-1-2
2	**19**	G-A	Signal peptide	Val-Asp	59–185
3	25	G-T	Signal peptide	Ala-Ser	154-90
4	28	A-G-C	Signal peptide	Lys-Glu-Gln	59-170-15
5	31	T-C	Signal peptide	Tyr-His	242-2
6	34/**35**	G/A-A/A-G/G-A/G	Signal peptide	Asp-Asn-Gly-Ser	139-11-91-3
7	**46**/**47**	G/A-G/T-A/T-T/A-G/G	Signal peptide	Asp-Val-Ile-Tyr-Gly	57-74-7-91-15
8	79/81	T/T-C/G	10	Tyr-Gln	129-115
9	88/89	C/A-A/A-C/G	13	Gln-Lys-Arg	174-68-2
10	107	G-A-C	19	Gly-Asp-Ala	224-2-18
11	118	C-G-T	23	Leu-Val-Leu	62-66-116
12	**125**	A-G	25	Lys-Arg	219-25
13	**127**	C-G	26	Leu-Val	242-2
14	163	C-G	38	Leu-Val	180-64
15	**190**	A-G-C	47	Tyr-Asp-His	66-173-5
16	196	A-G	49	Ser-Gly	242-2
17	211	T-C	54	Cys-Arg	242-2
18	215/216	A/T-G/T-A/G-G/G	55	Asp-Gly-Glu-Gly	66-166-1-11
19	217	G-A	56	Ala-Thr	242-2
20	227	A-G	59	Asn-Ser	68–176
21	238/239	A/G-A/A-G/A	63	Ser-Asn-Asp	222-16-6
22	251	A-G	67	Glu-Gly	242-2
23	**259**/260	G/C-G/T-A/T	70	Ala-Val-Met	80-151-13
24	262/**263**	C/A-T/C-T/A	71	Gln-Ser-*	156-81-7
25	287	C-A	79	Ala-Glu	225-19
26	295	C-A	82	Thr-Lys	93–151
27	314	T-C	88	Val-Ala	241-3
28	320	T-C	90	Leu-Pro	242-2
29	343	G-A	98	Val-Ile	236-8
30	350	A-G	100	Lys-Arg	128-115
31	364	A-G	105	Ile-Val	129-114
32	380	G-A	110	Gly-Asp	72–172
33	382	A-G	111	Arg-Gly	70–174
34	391	A-G	114	Ile-Val	70–174
35	394	T-C	115	Cys-Arg	242-2
36	401	A-G-T	117	Asp-Gly-Val	183-60-1
37	409	A-G	120	Thr-Ala	68–176
38	416	G-A-C	122	Arg-Gln-Pro	68-15-161

### Primer design and SNP mining of wild emmer wheat

Using the information from the 75 SNPs identified in the α-amylase inhibitor genes, 16 primers (combined with the reverse cloning primer, R, as SNP markers) were successfully designed to detect the SNPs in 205 accessions from 18 populations. The primers, with the SNP (bold letters) at the 3' end and an extra mismatched nucleotide (underline) on the third nucleotide from the end are listed in Table 2. A total of 14 SNPs were detected with the 16 SNP markers from position 19 to 288 of the α-amylase inhibitor gene, and the size of the amplified fragments ranged from 158 to 426 bp. The data was then organized in terms of genotypic frequencies ("0" or "1") to assess the population structure.

**Table 2:** Specific primers designed from SNPs in the dimeric α-amylase inhibitor genes

Primer	Sequences*	Fragment size	Temperature	Cycle
W19G	ATGCTCGTGGCGACACTC**G**	426	64	32
W24A	TCGTGGCGACACCCRTACC**A**	422	63	35
W35A	ACCCATAGCAGCCCAGTAC**AA**	412	62	35
W46A	CGAGTACGACGCATGGATC**A**	400	65	35
W47AT	AGTACGACGCATGGAGT**AT**	398	57	35
W125G	CTGTCGTCCATTGCTTA**G**	319	62.5	35
W127G	CTGTCGTCCATTGCTGAGG**G**	319	62.5	35
W190C	CTGCTGCCAGCAGCTCGGC**C**	256	57	35
W195T	TGCCAGCAGCTCGCCGACAT**T**	252	51	35
W207T	ACATCAGCGAGTGGAG**T**	236	60.5	30
W259A	CATGTATAAGGAGCATGTC**A**	187	61	35
W263C	TATAAGGAGCATGGCGTC**TC**	183	62	30
W263A	TATAAGGAGCATGGCGTC**TA**	183	65	35
W276A	GCGTGTCGGAGGGACAGTC**A**	170	60	35
W288CG	GACAGGCRGGGACAGG**A**A**CG**	158	66	30
W288AG	GACAGGCGGGGACAGG**A**A**AG**	158	63.5	30

*SNP positions were on the 3' end of the primers and are identified in bold letters. Extra mismatched nucleotides were also incorporated in the primers (underlined).

There were only 5 and 2 accessions from Yehudiyya and Achihood, respectively. Thus, the data for Yehudiyya and Achihood were not used in further analyses. Positive fragment frequency for each primer in the 16 populations is listed Additional file 1.

### Genetic diversity and distance of α-amylase inhibitor genes

Some genetic parameters of the 16 populations of wild emmer wheat are summarized in Table 3. The proportion of polymorphic loci *P *(5%), the expected heterozygosity *He*, and Shannon's information index of the 16 populations of wild emmer wheat were 0.887, 0.404, and 0.589, respectively. The values of *He *ranged from 0.182 to 0.437, and the population of Kokhav Hashahar had the highest value of *He *(0.437), followed by the population of Rosh-Pinna, whereas the population from Daliyya was characterized by the lowest *He *value of 0.182.

**Table 3:** Genetic diversity of wheat dimeric α-amylase inhibitor genes, based on SNPs in 16 populations of wide emmer wheat.

Population	No.	Sample Size	Polymorphic per population *P *^c^	Genetic diversity *He *^d ^(SE)	Shannon's information index^e ^(SE)
Mt. Hermon	1	9	1.000	0.374 (0.137)	0.550 (0.164)
Qazerin	5	12	1.000	0.414 (0.107)	0.600 (0.122)
Gamla	8	12	0.938	0.315 (0.169)	0.474 (0.219)
Rosh-Pinna	9	11	1.000	0.430 (0.097)	0.618 (0.110)
Tabiha	11	22	1.000	0.407 (0.083)	0.594 (0.091)
Mt. Gilboa	16	13	0.938	0.334 (0.155)	0.499 (0.201)
Mt. Gerizim	17	14	0.875	0.334 (0.197)	0.487 (0.259)
Gitit	18	13	0.875	0.338 (0.184)	0.493 (0.248)
Kokhav Hashahar	19	9	1.000	0.437 (0.088)	0.625 (0.098)
J'aba	23	12	0.938	0.385 (0.133)	0.561 (0.176)
Amirim	24	12	0.875	0.337 (0.181)	0.493 (0.248)
Nahef	25	9	0.625	0.217 (0.217)	0.322 (0.303)
Beit-Oren	28	16	0.875	0.308 (0.173)	0.460 (0.235)
Daliyya	29	8	0.500	0.182 (0.200)	0.273 (0.292)
Bat-Shelomo	30	13	0.875	0.291 (0.180)	0.438 (0.241)
Givat-Koach	33	13	0.875	0.295 (0.182)	0.442 (0.245)
Mean		198	0.887	0.404 (0.102)	0.589 (0.118)

The genetic distances (*D*) were calculated for comparisons of all 16 populations based on the positive fragment of SNP markers among all population pairs (see Additional file 2). The highest genetic distance (0.263) was obtained between populations of Kokhav Hashahar and Daliyya, whereas the most related populations were Qazzrin and Gamla with a genetic distance of 0.017. However, lower *D *values (< 0.050) were observed between some populations from different areas, and, for the most part, the estimates of *D *value were geographically independent. Large genetic distances and sharp genetic differentiation over long geographic distances could be found. For example, Kokhav Hashahar in southern Israel had higher *D *values with the populations from Gamla (0.221), Nahef (0.247), Beit-Oren (0.215), Daliyya (0.263), and Bat-Shelomo (0.224) in northeast Israel.

### Principle components & multiple regression analysis of environmental variables and SNPs

To assess if some of the ecological factors are correlated to each other, principle components analysis (PCA) was carried out using 23 ecological factors as variables. A combination of the first four components could give us a high cumulative percent (88.81%) according to the eigenvalues of the correlation matrix (see Additional file 3A; 4), which could be used to explain the ecological associations. The main ecological factors of the first component were Rd and Ev (two water availability factors) that could give 40.60% eigenvalues, and the second component could give 28.66% eigenvalues (see Additional file 3). And from PCA analysis, it was known that the accessions from Mt. Hermon were affected the most by ecological factors (see Additional file 3C).

After analyzed of the factors by projection of the variables on the factor-plane (see Additional file 4) and consulted the correlations of these factors (see Additional file 5), 11 independent ecological factors were chosen. And then, multiple regression analysis was done using these 11 factors to investigate the relationship between environmental variables and SNPs.

The geographical, temperature, water, and solar radiation factors in Table 4, singly or in combination, explained a significant proportion of the diversity in the SNPs (Table 5). The best variable predictors of *P*, *He*, and Shannon's information index, significantly explaining 0.264 – 0.355 of the variance, was the water availability factor Hu-an. The combination of three variable predictors accounting for geographic and water availability factors Hu-an, Ev, and Lt (mean annual humidity, mean annual evaporation and latitude) accounted significantly (p < 0.05) for about 0.55 of the genetic diversity. The addition of a fourth variable predictor, Rad (total solar radiation per year), or Sh (mean number of Sharav days) to the first three factors accounted for approximately 0.65 of the diversity (significant at p < 0.05) (Table 5).

**Table 4:** The eco-geographical background of populations in this study

No.	Population*	N	Ln	Lt	Al	Tm	Ta	Tj	Td	Tdd	Rn	Rd	Hu 14	Hu an	Dw	Sh	Th	Trd	Ev	Sz	Ma	So	Rv	Rr	Rad
1	Mt. Hermon	9	35.73	33.30	1300	11	21	3	18	6	1400	66	48	60	60	80	-	0	150	2	1	1	30	20	185
5	Qzzrin	12	35.67	32.99	350	18	26	10	16	12	530	50	43	58	58	50	-	60	155	3	5	5	39	26	189
7	Yehudiyya	5	35.70	32.93	200	19	27	11	16	12	550	47	42	58	58	50	-	100	160	3	5	5	38	25	189
8	Gamla	12	35.74	32.88	200	19	26	9	17	12	470	50	43	58	58	50	-	60	155	3	5	5	39	26	-
9	Rosh-Pinna	11	35.52	32.95	700	18	25	9	16	10	697	50	48	58	50	75	-10	35	150	3	5	1	35	22	184
11	Tabiha	22	35.53	32.90	0	24	32	15	17	10	436	45	45	57	58	60	-30	120	160	3	5	5	39	25	188
16	Mt. Gilboa	13	35.42	32.50	150	21	28	12	16	12	400	43	43	58	40	60	-30	160	165	2	3	1	34	24	189
17	Mt. Gerizim	14	35.28	32.20	800	17	23	8	15	9	700	45	45	60	42	-	10	0	155	2	3	1	38	25	186
18	Gitit	13	35.40	32.10	300	21	29	13	16	12	360	39	39	55	25	-	-25	100	170	2	3	1	38	24	195
19	Kokhav Hashahar	9	35.34	31.95	600	20	28	12	16	12	400	45	45	59	30	80	-20	25	165	2	3	1	38	22	195
23	Jaba	12	35.08	31.67	660	17	25	9	15	9	500	49	49	62	57	90	-20	30	155	2	3	1	35	21	186
24	Amirim	12	35.45	32.93	600	15	24	8	16	8	850	48	48	60	53	85	0	13	153	2	2	1	35	23	182
25	Nahef	9	35.32	32.93	275	15	24	8	15	9	670	49	49	62	57	62	10	3	155	1	2	1	33	22	181
26	Achihood	2	35.17	32.91	25	19	26	11	15	10	590	53	53	65	62	40	-5	20	148	1	2	1	30	21	180
28	Beit-Oren	16	35.03	32.73	400	17	24	11	13	8	700	59	59	69	80	41	5	0	142	1	2	1	25	19	183
29	Daliyya	8	35.06	32.59	200	19	26	12	14	11	670	57	57	67	78	50	-10	100	160	1	2	2	25	20	181
30	Bat-Shelomo	13	35.02	32.60	75	20	26	13	13	10	650	58	58	68	77	40	-10	30	150	2	2	2	24	20	182
33	Givat-Koach	13	34.92	32.03	75	20	26	12	14	12	540	50	50	64	65	42	-20	105	160	1	2	1	32	26	180

Population numbers and ecological factors definitions was according to Nevo and Beiles 1989.

* Populations Yehudiyya (7) and Achihood (26) do not have enough accessions to do further statistical analysis.

Symbols of Variables

i. **Geographical**: Ln = Longitude; Lt = latitude; Al = altitude

ii. **Temperature**: Tm = mean annual temperature; Ta = mean August temperature; Tj = mean January temperature; Td = seasonal temperature difference; Tdd = day-night temperature difference; Trd = mean number of tropical days (tropical days is defined by meteorologists check internet or atlases); Sh = mean number of Sharav days, i.e., hot and dry days

iii. **Water availability**: Rn = mean annual rainfall; Rd = mean number of rainy days; Hu-an: = mean annual humidity; Hu-14 = mean humidity at 14:00 h; Dw = mean number of dewy nights in summer; Th = Thornthwaite's moisture index (indicator of the supply of water in an area relative to the demand under prevailing climatic conditions); Ev = mean annual evaporation; Rv = mean inter-annual variability of rainfall; Rr = mean relative variability of rainfall

iv. **Edaphic**: So = soil type: 1 = terra-rossa (t.r.); 2 = rendzina; 5 = basalt

v. **Biotic**: Ma = marginality (A measure of the ecological distance and direction by which the mean of the species distribution differs from the mean of the global distribution): 1 = North margin, 2 = West margin, 3 = Southeast margin, 5 = central population; Sz = estimate of population size: 1 = small (from a dozen to few hundred plants), 2 = intermediate, 3 = large

vi. **Solar radiation**: Rad = total solar radiation per year

**Table 5:** Coefficient of multiple regressions of genetic indices and allele frequencies and environmental variables in 16 populations of wild emmer wheat as independent variables. *** = p < 0.001; ** = p < 0.01; * = p < 0.05; @ = p < 0.10; ns = p > 0.10. The definitions of factors were in Table 4.

Genetic indices	Stepwise model by ecogeographical variables								
	
	STEP1	STEP2	STEP3	STEP4	STEP5	STEP6	STEP7	STEP8	STEP9
*P*	Hu-an 0.264**	Ev 0.422**	Lt 0.533*	Sh 0.641*	Rd 0.714**	Rad 0.790***	Tm 0.828**		
*H*	Hu-an 0.355***	Ev 0.465**	Lt 0.599*	Rad 0.678*	Sh 0.778**	Ln 0.862ns	Th 0.912@	Rd 0.935ns	Rr 0.953@
Shannon's Index	Hu-an 0.345***	Ev 0.463**	Lt 0.593*	Rad 0.659*	Sh 0.773**	Td 0.864***	Tm 0.906**	Ln 0.941ns	
Allele Frequency									
W19G	Td 0.226ns	Ln 0.371ns	Rr 0.449ns	Lt 0.524ns					
W24A	Rd 0.589***	Rad 0.706**	Tm 0.748@	Sh 0.836ns	Td 0.864@				
W35A	Td 0.190ns	Rr 0.421ns	Lt 0.582@	Sh 0.689*	Rad 0.783*	Th 0.809**			
W46A	Ev 0.148ns	Rad 0.243ns	Rr 0.347ns						
W47AT	Th 0.179***								
W125G	Lt 0.197@	Rr 0.275@	Ev 0.475ns	Rd 0.569ns	Tm 0.624ns	Td 0.763ns			
W127G	Hu-an 0.473**	Tm 0.586*	Rad 0.665@	Rd 0.725ns					
W190C	Rd 0.217*	Sh 0.287ns							
W195T	Rad 0.482***	Rd 0.520***	Lt 0.584ns						
W207T	Td 0.168*	Lt 0.304ns	Rad 0.366ns	Sh 0.502ns					
W259A	Rad 0.294ns	Lt 0.406ns	Th 0.592*	Tm 0.640*	Ev 0.740**	Sh 0.882**	Td 0.904**	Rr 0.921**	
W263TC	Sh 0.181***								
W263TA	Td 0.235*	Lt 0.438ns	Ev 0.610*	Sh 0.748**	Ln 0.771ns	Rd 0.799ns	Tm 0.845ns	Rr 0.871ns	
W276A	Ev 0.114ns								
W288CG	Td 0.192ns	Rr 0.419ns	Rd 0.520ns	Th 0.572ns	Sh 0.615@	Tm 0.683*			
W288AG	Ln 0.187@	Ev 0.382@	Rd 0.501ns	Rr 0.548ns					

SNPs could be classified into several categories in terms of their prime ecogeographical predictors. The best single variable predictors of SNP marker allele frequencies were: (1) water (Hu-an, Rr, Th, Rd, Ev): W24A, W46A, W47AT, W127G, W190C, W276A; (2) temperature (Td, Tm, Sh): W19G, W35A, W207T, W263TC, W263TA, W288CG; (3) geographic (Ln, Lt): W125G, W288AG; and (4) solar radiation (Rad): W195T; W259A. It was obvious that water factors were the best variable predictors, singly or in combination, with other ecological factors (Table 5).

### Spearman rank correlations of SNP positions with environment

The average of genetic indices (*P*, *He*, and Shannon's information index) and *He *of each of the SNP positions with ecogeographical variables appear in Table 6. We recorded the ecological variables for the populations. The *P*, *He*, and Shannon's information index were negatively correlated with the three water factors: mean annual humidity (Hu-an), mean humidity at 14:00 h (Hu-14), and mean number of dew nights in summer (Dw). However, they correlated positively with other factors: latitude (Ln), seasonal temperature difference (Td), estimate of population size (Sz), marginality (Ma), mean inter-annual variability of rainfall (Rv), and total solar radiation per year (Rad). The correlation matrix between *He *in the SNPs and geographic variables contained 30 significant (p < 0.05) correlations. Five SNPs (e.g., W47AT, W125G, W127G, W263TA, and W288AG) positively correlated with Sz (rs = 0.556–0.687), and four SNPs (e.g., W19G, W47AT, W127G, and W288AG) positively correlated with Ln (rs = 0.508–0.567). Four SNPs: W24A, W127G, W190C, and W263TA negatively correlated with two water factors, Hu-an or Rn (mean annual rainfall or mean annual humidity) or both of these factor (Table 6).

**Table 6:** Spearman rank correlations of genetic indices and the genetic diversity of each SNP sites.

	Ln	Al	Td	Rd	Hu-14	Hu-an	Dw	Sh	Sz	Ma	Rv	Rr	Rad
Shannons' infor. index	0.568*		0.629**		-0.532*	-0.644**	-0.518*		0.691**	0.593*	0.596*		0.724**
*He*	0.559*		0.622*		-0.528*	-0.647**	-0.521*		0.691**	0.585*	0.629**		0.716**
*P*	0.665**		0.705**			-0.590*			0.762**	0.565*	0.518*		0.524*
***He***													
W19G	0.535*		0.580*										
W24A				-0.555*		-0.542*	-0.626**	0.499*					
W47AT	0.508*								0.556*				
W125G									0.587*				
W127G	0.567*		0.656**	-0.513*	-0.729**	-0.824**	-0.623**	0.687**	0.619*	0.648**		0.689**	
W190C				-0.508*									
W259A		0.529*											0.577*
W263TC													0.534*
W263TA						-0.503*			0.649**	0.614*	0.516*	0.521*	
W288AG	0.517*								0.645**				

** = p < 0.01; * = p < 0.05

## Discussion

### SNPs in the α-amylase inhibitor genes

In sequence comparisons, the 244 dimeric α-amylase inhibitor genes from wild emmer wheat, had a high level of similarity, indicating that the primary structure of these genes was similar to those of known dimeric α-amylase inhibitors 0.19 (WDAI-0.19) and 0.53 (WDAI-0.53). The predicted protein sequence of the 244 cloned α-amylase inhibitor genes from wild emmer wheat showed the presence of 10 Cys, which were the amino acids most important to the structure and function of the mature protein [31]. Changes in structure of α-amylase inhibitor proteins would affect their specificity and activity against different mammalian and insect α-amylase [32]. A comparison of sequence between members of the α-amylase inhibitors 0.19 group indicated that not only the 10 Cys residues were of importance, but also Asp110, Lys116, Asn29, Glu35, Ser94, Leu90, Trp51, His47, and Gln13 were important to form the structure of those inhibitors [33]. Most of the SNPs did not occur at highly conserved positions, which ensures that the α-amylase inhibitors keep their correct 3D structure to combine with the α-amylase. However, Gln13, His47, Ser49, Leu90, Val105, and Asp110 were changed by SNPs in some of the cloned α-amylase inhibitor genes (Table 1). It is noteworthy that only the α-amylase inhibitors from the D genome of *Ae. tauschii *and common wheat, which were closely related to inhibitor 0.19, had the His47 [30], whereas the His47 was replaced by Asp or Tyr in 98% of the inhibitor genes from wild emmer wheat.

### Genetic diversity of the α-amylase inhibitor genes in wild emmer wheat

Genetic diversity of the α-amylase inhibitor genes of 198 wild emmer wheat accessions from 16 populations in Israel were revealed by 16 SNP markers. Individual accessions from different populations could not be distinguished clearly by the sequences of their α-amylase inhibitor genes; whereas, using the SNP-specific primers, all wild emmer wheat populations were distinguishable, even within closely related populations originating in proximate geographic locations (Table 3). Our results demonstrated that the polymorphism of α-amylase inhibitor genes in wild emmer wheat correlated with the ecogeographic distribution of the accessions. The results suggest that the gene was subjected to strong natural selection. The observations were consistent with previous results obtained with high- and low-molecular-weight glutenin subunits, which are also seed storage proteins [34-36]. In other studies, DNA diversity of glutenin subunits was shown to be correlated to environmental factors and variation [34].

The genetic diversity profiles in this study were compared with earlier allozyme studies [1], RAPD loci [37], and with the microsatellite studies [38] in wild emmer wheat populations. Although the SNP markers in the protein-coding genes yielded lower values of diversities than other methods, the results in this study were able to reveal the correlations of SNP variations in specific functional genes with ecological factors.

Central populations used in this study were collected in warm, humid environments on the Golan Plateau and near the Sea of Galilee. Marginal steppic populations were collected across a wide geographic area on the northern, eastern, and southern borders of wild emmer distribution involving hot, cold, and xeric peripheries, while marginal mesic (Mediterranean) populations were collected from the western border of wild emmer distribution [1,39]. The present study included 198 accessions collected from 16 different sites in Israel and covered a wide range of ecogeographical conditions across the distribution range of the species. Specific SNP positions detected in the α-amylase inhibitor genes were found to be highly effective in distinguishing genotypes and populations of wild emmer wheat originating from diverse ecogeographic sites in Israel. High levels of polymorphic loci (*P*), expected heterozygosity (*He*), and Shannon's information index (Table 3) with high genetic distance values between populations could be found (see Additional file 2). These results suggest that the genetic variation at these SNP positions in the dimeric α-amylase inhibitor genes was somewhat ecologically determined for these populations.

### Genetic distance versus geographical distance

**Figure 2:**
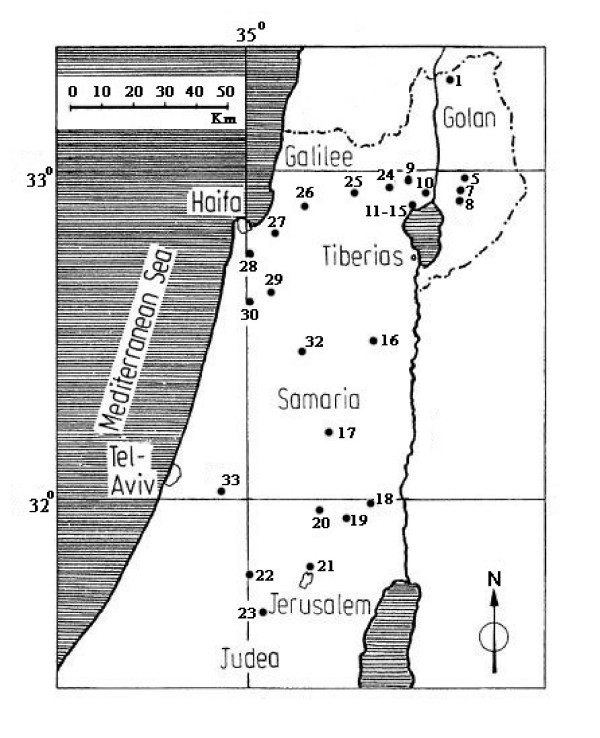
**Geographic distribution of the tested populations of wild emmer wheat.** The numbered populations are according to Nevo and Beiles (1989) [1] and details about the populations can be found in Table 4.

The relationship between SNPs' genetic distance and geographical distance was investigated, and it was found that the estimates of genetic distance (*D*) were geographically independent, as was previously found for allozymes, RAPD loci, and microsatellite analyses [1,37,38]. Quite often it is easier to find a greater genetic difference between proximal populations than among populations that are far apart. This was clearly demonstrated by local short transects of different soil types at Tabigha [40] and by the micro-differences of sun-shade differentiation at Yehudiyya [41]. Sharp genetic divergence (large *D*) over very short geographic distances against small genetic divergence (small *D*) between large geographically distances were observed in wild emmer populations (see Additional file 2). For example, it was shown that the genetic distance obtained between the population at Gitit and the population at Kokhav Hashahar (located only about 10 km apart), with *D *= 0.1513, was 2.66 times higher than the genetic distance between the population at Mt. Hermon and the population at J'aba (separated by 160 km, with *D *= 0.0569). In other words, the distance between the first 2 populations was 1/16 of the distance between the second 2 populations (Figure 2 and Additional file 2).

The genetic structure of wild emmer wheat populations in Israel is mosaic [36]. This patchy genetic distribution appears to reflect the underlying ecological heterogeneity at both micro- and macro-scales [1,37,38,40,41]. Thus the higher polymorphisms and genetic variations of dimeric α-amylase inhibitor genes within and between populations could be explained by natural selection.

### Ecological genetics of SNPs in dimeric α-amylase inhibitor genes

Natural populations of wild emmer are highly polymorphic in morphological characters, as well as for various economically important traits [3,5,34]. Although major collection areas such as Mt. Hermon, Rosh Pinna, Gamla, Bat-Shelomo, and Tabigha are at similar longitude and latitude, they differ significantly in altitude. These locations, for example, are respectively at 1300, 700, 200, 75, and 0 m above sea level (Table 4). Along with these features, several other environmental factors differ for these locations [1,39].

In this study, the mean number of *P*, *He*, and Shannon's information index were negatively correlated with the three water factors and positively correlated with the other six factors (Table 6). It was noteworthy that the significant ecological factors (Ln, Ta, Td, Hu-14, Hu-an, Dw, and Rv), revealed by a Spearman rank correlations matrix between allozyme and climate, were similar to the results in this study [1]. This similarity might be because the correlation between ecological factors and coding sequences or proteins (allozyme) is different from the non-coding sequences. Moreover, the correlation between photosynthetic performance and ecogeographical variables indicated that ecological factors, e.g., sharav (Sh), dewy nights (Dw), radiation (Rad), rainy days (Rd), altitude (Al), and latitude (Lt) were distinctly correlated with photosynthetic factors [42]. Photosynthetic efficiency needs specific ecological factors, especially light.

In this study, the SNP variations showed significant correlations with ecological factors (Table 5; Table 6). Geographical, temperature, water availability, edaphic, biotic and solar radiation factors (Sz, So, Rad, Al, Rn, Lt, Sh, Rv, Ln, Td, Hu-14, Hu-an, Dw, and Ma), singly or in combination, explained a significant proportion of the diversity in the SNPs of α-amylase inhibitor genes. The association of these factors with SNPs was similar to the association of latitude/altitude with RAPD and microsatellite diversity [37,38]. It could be explained by the change in ecological factors, i.e., Al, the sharp gradient of climatic conditions from north to south in Israel, with increasing temperatures and decreasing water availability towards the semiarid zones in southern Israel. Also, the ecological factors used in this study were not representative of all the possible components involved in the determination of the real climate.

The SNPs that could determine the amino acid changes in the mature protein of α-amylase inhibitors were of great importance. Six specific primers (W125G, W127G, W190C, W259A, W263TC, and W263TA) were designed, based on the SNPs at five positions associated with amino acids changes (Table 1). It was shown that these SNPs were significantly correlated with water availability factors (Rd, and Hu-an), temperature factors (Sh, and Td), geographical factors (Ln, Al, and Lt), and solar radiation (Rad) better than the other factors (Table 5 and Table 6). Environmental stress can greatly influence plant susceptibility to herbivores and pathogens, and drought stress can promote outbreaks of fungal diseases and plant-eating insects [43,44]. Louda and Collinge (1992) reported guild-specific insect responses following soil water manipulations, and Larssou (1989) has clearly articulated why the actual response of insect herbivores to plant stress should be feeding-guild specific [45,46]. The results in this study indicated that water availability is the main factor that could affect the dimeric α-amylase inhibitor genes and, thus, the concordance between insect and plant. Recently, based on SNP analysis, highly significant correlations were also found between diversity at the barley *Isa *locus (coding for a bi-functional α-amylase/subtilisin inhibitor) and key water variables (evaporation, rainfall, and humidity) plus latitude [47]. The soil fungi may influence the survival of wild barley seed in soil and the subsequent establishment of plant populations. The higher diversity of soil fungi in dry environments may select for a higher diversity of defense proteins encoded by the *Isa *locus in the seed [47].

The herbivore insect and the level of herbivore pressure may vary with ecological factors, so that the wheat is under different herbivore-related selection pressures at each site. Different environmental pressures at each site directly related to the climate, but the wheat alpha-amylase inhibitors responded indirectly to those factors. There might be some evolutionary mechanisms that underlie the differences in diversity of α-amylase inhibitors and water factors. Historical events may have given rise to diversity patterns that correlate coincidentally with the ecogeographical variables tested in this study. However, probability would suggest it is far more likely that the variation in genetic diversity of this gene between populations is a product of selective forces. Selection pressure at this locus is likely to be caused by insects.

## Conclusion

The populations of wild emmer wheat showed great diversity in dimeric α-amylase inhibitors, both between and within populations. We suggest that SNP markers are useful for the estimation of genetic diversity of protein-coding genes in wild emmer wheat. These results show significant correlations between SNPs in the α-amylase inhibitor genes and ecological diversities. Ecological factors, singly or in combination, explained a significant proportion of the variations in the SNPs, and the SNPs could be classified into several categories as ecogeographical predictors. A sharp genetic divergence (large *D*) over very short geographic distances against small genetic divergence (small *D*) between large geographical distance was found in wild emmer populations. It was suggested that the SNPs in the α-amylase inhibitor genes were subjected to natural selection, and ecological factors had an important evolutionary role in gene differentiation at the gene loci.

## Methods

### Plant material and ecological background of wild emmer wheat

Wild emmer wheat is a tetraploid and predominantly self-pollinated wheat, which is distributed over the Near East Fertile Crescent (Israel, Jordan, Lebanon, Syria, eastern Turkey, northern Iraq, and western Iran) [48]. The center of distribution and diversity of emmer wheat was found in the catchment area of the upper Jordan Valley (Golan Heights, eastern Upper Galilee Mountains, etc.) in Israel and its vicinity [1]. Wild emmer wheat covers wide ranges of eco-geographical conditions in Israel. However, towards their marginal and peripheral areas, both in Israel and Turkey, wild emmer wheat became semi-isolated or isolated, and smaller in size. This distributional pattern has a dramatic effect on their population genetic structure and differentiation [1]. Individual plants of emmer wheat were collected at random, at least 1 m apart, from populations differing in major ecological properties. These collection sites and populations have been described in detail elsewhere [1,39]. The genotypes used for the present study are conserved in the cereal gene bank of the Institute of Evolution, University of Haifa.

In this study we examined 205 *T. dicoccoides *accessions representing 18 populations collected from various locations in Israel, which represent a wide range of ecological conditions of soil, temperature, altitude, and water availability. The populations used in this study, along with their geographic origin and climatic conditions, are listed in Table 4. A full description of these populations was reported in Nevo *et al*. [1,39], and the map location of these populations were provided in Figure 2.

### DNA isolation and PCR amplification

Ten seeds of each accession were germinated in the dark at room temperature. Genomic DNA was extracted from plant leaves at about 2 weeks of age with a modified CTAB protocol, as described in Murray and Thompson [49]. Two primers, F (5'-CTATGTATGCTCGTGGCGAC-3') and R (5'-ACTCATTT/CGCTTGACTAGGC-3'), were used to amplify the gene coding sequences of dimeric α-amylase inhibitors [30]. PCR amplification was performed with PTC-240 cycler (Bio-Rad) in 50 μL volume, which consisted of about 100 ng of genomic DNA, 100 μM of each dNTPs, 1 μM of each primers, 1U *Taq *polymerase, 1.5 mM Mg^2+^, and 1×PCR buffer. The cycling parameters were 95°C for 5 min to pre-denature, followed by 35 cycles of 95°C for 1 min, 60°C for 30 sec and 72°C for 1 min, and a final extension at 72°C for 5 min.

### Sequence analysis of α-amylase inhibitor

Amplification products were separated in 2% agarose gels. The desired DNA fragments were recovered from gels and ligated to the pBluescript SK (+) T-vector plasmid (Stratagene), and then the positive clones were screened and sequenced. The analysis of full-length sequence and the construction of subsequent nucleotide sequence were carried out under DNAman 5.2.2 [50], and the multiple sequence alignment software Clustal W [51] was used for the SNP assessment. The α-amylase inhibitor ORFs were translated into amino acid sequences using the ORF Finder program at the NCBI [52]. The polymorphic positions were used instead of all of the mutation positions, including the positions with change that observed only once in the dataset, in the subsequent analysis.

### Specific primer design and analysis of SNP

Polymorphic positions were identified by MEGA version 3.1 [53]. Sixteen specific PCR forward primers (combined with the cloning reverse primer R), were designed based on the alignments of dimeric α-amylase inhibitor gene sequences obtained from wild emmer wheat (Table 2). The SNPs were positioned at the 3'-end of the primers, based on the fact that a 3' mismatch makes PCR more specific at the selected annealing temperature [54,55]. The power of the oligonucleotide for allele discrimination was enhanced by introducing an artificial mismatch at the 3'-terminal base [56]. Sequences for the specific primers for dimeric α-amylase inhibitor genes and the basic cycling conditions are listed in Table 2. PCR was performed on genomic DNAs from all accessions of the 18 populations.

### Data acquisition and analysis

The gels were scored for the presence or absence of bands that showed a reproducible pattern among genotypes, and for each band with a SNP position with two alternative alleles: present (1) or absent (0). For wild emmer wheat, which is a self-pollinating species with a quite limited rate of outcrossing (estimated *t *approximately 0.005), we assumed 100% homozygosity. The identification of 16 SNP positions led to the construction of a 198 accessions (two populations with less than 8 accessions were not used in this analysis) × 16 loci data matrix, which was analyzed for diversity within and between populations.

POPGENE 1.32 [57] was used to compute genetic polymorphism (*P*), expected heterozygosity (Nei's gene diversity) (*He*), and Shannon's information index (*I*) for each SNP position and population. Spearman rank correlation coefficients were used to assess differences in genetic indices *P*, *He*, and Shannon's information index and climatic variables in 16 populations. STATISTICA version 6.0 [58] was used to do the PCA analysis and conduct stepwise multiple regression (MR). Multiple regression analysis was conducted to test the best predictors of *P*, *He*, and Shannon's information index in the 16 populations using these genetic indices as dependent variables and the eco-geographic factors as independent variables at each of the polymorphic SNP loci.

## Authors' contributions

WJR designed and carried out the experiment and wrote the manuscript. WYM designed the experiment, formulated the questions, and contributed to writing the manuscript. LXY participated in the experiment. YZH carried out sequence alignment analysis. BB contributed to the data analysis and writing. EN formulated the questions, retrieved and analyzed the data, and planned the experiments. ZYL planned the study and participated in the design of the experiments.

## Supplementary Material

Additional file 1 Positive fragment and the frequency of each primer in 16 Population of wild emmer wheat. This data showed the frequency of each specific primer in the 16 populations calculated by POPGENE 1.32. Click here for file

Additional file 2 Nei's genetic distance of the inhibitor genes in the 16 populations. This data showed the genetic distances (*D*) based on the positive fragment of SNP markers among all population pairs. Click here for file

Additional file 3 Principal components analysis. This data showed the eigenvalues of correlation matrix, eigenvectors and factor coodinates of 16 populations. Click here for file

Additional file 4 Principal components analysis of the ecological factors. This data showed the projection of the variables on the ecological factor-plane and eigenvalues of correlation matrix. Click here for file

Additional file 5 Correlations of the Factors. This data showed the correlation of the 20 ecological factors that separated into four groups. Click here for file
