# Supplementary material for: Molecular evolution of dimeric α-amylase inhibitor genes in wild emmer wheat and its ecological association
Source: BMC Evol Biol. 2008 Mar 24;8:91. doi: 10.1186/1471-2148-8-91 (PMC2324104; doi:10.1186/1471-2148-8-91)
Supplement: Additional file 1 — Positive fragment and the frequency of each primer in 16 Population of wild emmer wheat. This data showed the frequency of each specific primer in the 16 populations calculated by POPGENE 1.32. [file 1471-2148-8-91-S1.doc]

Additional file 1. Positive fragment and the frequency of each primer in 16 Population of wild emmer wheat

| No. | Population | N | W19G | | | W24A | | | W35A | | | W46A | | |
| --- | --- | --- | --- | --- | --- | --- | --- | --- | --- | --- | --- | --- | --- | --- |
|  |  |  | "+" | "%" | Frequncy | "+" | "%" | Frequncy | "+" | "%" | Frequncy | "+" | "%" | Frequncy |
| 1 | Mt. Hermon | 9 | 7.00 | 77.78 | 0.53 | 2.00 | 22.22 | 0.12 | 7.00 | 77.78 | 0.53 | 4.00 | 44.44 | 0.25 |
| 5 | Qzzrin | 12 | 8.00 | 66.67 | 0.42 | 7.00 | 58.33 | 0.35 | 3.00 | 25.00 | 0.13 | 7.00 | 58.33 | 0.35 |
| 7 | Yehudiyya | 5 | 4.00 | 80.00 |  | 1.00 | 20.00 |  | 2.00 | 40.00 |  | 1.00 | 20.00 |  |
| 8 | Gamla | 12 | 6.00 | 50.00 | 0.29 | 3.00 | 25.00 | 0.13 | 1.00 | 8.33 | 0.04 | 3.00 | 25.00 | 0.13 |
| 9 | Rosh-Pinna | 11 | 8.00 | 72.73 | 0.48 | 6.00 | 54.55 | 0.33 | 4.00 | 36.36 | 0.20 | 6.00 | 54.55 | 0.33 |
| 11 | Tabiha | 22 | 18.00 | 81.82 | 0.57 | 21.00 | 95.45 | 0.79 | 8.00 | 36.36 | 0.20 | 14.00 | 63.64 | 0.40 |
| 16 | Mt. Gilboa | 13 | 5.00 | 38.46 | 0.22 | 9.00 | 69.23 | 0.45 | 5.00 | 38.46 | 0.22 | 5.00 | 38.46 | 0.22 |
| 17 | Mt. Gerizim | 14 | 3.00 | 21.43 | 0.11 | 10.00 | 71.43 | 0.47 | 1.00 | 7.14 | 0.03 | 7.00 | 50.00 | 0.29 |
| 18 | Gitit | 13 | 10.00 | 76.92 | 0.52 | 12.00 | 92.31 | 0.72 | 2.00 | 15.38 | 0.08 | 7.00 | 53.85 | 0.32 |
| 19 | Kokhav Hashahar | 9 | 7.00 | 77.78 | 0.53 | 6.00 | 66.67 | 0.42 | 8.00 | 88.89 | 0.67 | 8.00 | 88.89 | 0.67 |
| 23 | Jaba | 12 | 11.00 | 91.67 | 0.71 | 9.00 | 75.00 | 0.50 | 5.00 | 41.67 | 0.24 | 4.00 | 33.33 | 0.18 |
| 24 | Amirim | 12 | 6.00 | 50.00 | 0.29 | 6.00 | 50.00 | 0.29 | 1.00 | 8.33 | 0.04 | 7.00 | 58.33 | 0.35 |
| 25 | Nahef | 9 | 4.00 | 44.44 | 0.25 | 5.00 | 55.56 | 0.33 | 1.00 | 11.11 | 0.06 | 1.00 | 11.11 | 0.06 |
| 26 | Achihood | 2 | 2.00 | 100.00 |  | 1.00 | 50.00 |  | 2.00 | 100.00 |  | 2.00 | 100.00 |  |
| 28 | Beit-Oren | 16 | 8.00 | 50.00 | 0.29 | 3.00 | 18.75 | 0.10 | 1.00 | 6.25 | 0.03 | 6.00 | 37.50 | 0.21 |
| 29 | Daliyya | 8 | 4.00 | 50.00 | 0.29 | 4.00 | 50.00 | 0.29 | 2.00 | 25.00 | 0.13 | 3.00 | 37.50 | 0.21 |
| 30 | Bat-Shelomo | 13 | 4.00 | 30.77 | 0.17 | 5.00 | 38.46 | 0.22 | 3.00 | 23.08 | 0.12 | 5.00 | 38.46 | 0.22 |
| 33 | Givat-Koach | 13 | 6.00 | 46.15 | 0.27 | 6.00 | 46.15 | 0.27 | 2.00 | 15.38 | 0.08 | 10.00 | 76.92 | 0.52 |
|  |  |  | W47AT | | | W125G | | | W127G | | | W190C | | |
|  |  |  | "+" | "%" | Frequncy | "+" | "%" | Frequncy | "+" | "%" | Frequncy | "+" | "%" | Frequncy |
| 1 | Mt. Hermon | 9 | 8.00 | 88.89 | 0.67 | 2.00 | 22.22 | 0.12 | 1.00 | 11.11 | 0.06 | 1.00 | 11.11 | 0.06 |
| 5 | Qzzrin | 12 | 7.00 | 58.33 | 0.35 | 6.00 | 50.00 | 0.29 | 2.00 | 16.67 | 0.09 | 4.00 | 33.33 | 0.18 |
| 7 | Yehudiyya | 5 | 5.00 | 100.00 |  | 5.00 | 100.00 |  | 4.00 | 80.00 |  | 2.00 | 40.00 |  |
| 8 | Gamla | 12 | 9.00 | 75.00 | 0.50 | 7.00 | 58.33 | 0.35 | 1.00 | 8.33 | 0.04 | 2.00 | 16.67 | 0.09 |
| 9 | Rosh-Pinna | 11 | 9.00 | 81.82 | 0.57 | 7.00 | 63.64 | 0.40 | 3.00 | 27.27 | 0.15 | 2.00 | 18.18 | 0.10 |
| 11 | Tabiha | 22 | 20.00 | 90.91 | 0.70 | 13.00 | 59.09 | 0.36 | 6.00 | 27.27 | 0.15 | 12.00 | 54.55 | 0.33 |
| 16 | Mt. Gilboa | 13 | 12.00 | 92.31 | 0.72 | 2.00 | 15.38 | 0.08 | 2.00 | 15.38 | 0.08 | 2.00 | 15.38 | 0.08 |
| 17 | Mt. Gerizim | 14 | 14.00 | 100.00 | 1.00 | 3.00 | 21.43 | 0.11 | 2.00 | 14.29 | 0.07 | 6.00 | 42.86 | 0.24 |
| 18 | Gitit | 13 | 13.00 | 100.00 | 1.00 | 1.00 | 7.69 | 0.04 | 3.00 | 23.08 | 0.12 | 7.00 | 53.85 | 0.32 |
| 19 | Kokhav Hashahar | 9 | 8.00 | 88.89 | 0.67 | 2.00 | 22.22 | 0.12 | 3.00 | 33.33 | 0.18 | 8.00 | 88.89 | 0.67 |
| 23 | Jaba | 12 | 8.00 | 66.67 | 0.42 | 3.00 | 25.00 | 0.13 | 0.00 | 0.00 | 0.00 | 5.00 | 41.67 | 0.24 |
| 24 | Amirim | 12 | 11.00 | 91.67 | 0.71 | 8.00 | 66.67 | 0.42 | 1.00 | 8.33 | 0.04 | 9.00 | 75.00 | 0.50 |
| 25 | Nahef | 9 | 9.00 | 100.00 | 1.00 | 0.00 | 0.00 | 0.00 | 0.00 | 0.00 | 0.00 | 1.00 | 11.11 | 0.06 |
| 26 | Achihood | 2 | 2.00 | 100.00 |  | 0.00 | 0.00 |  | 0.00 | 0.00 |  | 2.00 | 100.00 |  |
| 28 | Beit-Oren | 16 | 14.00 | 87.50 | 0.65 | 8.00 | 50.00 | 0.29 | 0.00 | 0.00 | 0.00 | 3.00 | 18.75 | 0.10 |
| 29 | Daliyya | 8 | 8.00 | 100.00 | 1.00 | 0.00 | 0.00 | 0.00 | 0.00 | 0.00 | 0.00 | 2.00 | 25.00 | 0.13 |
| 30 | Bat-Shelomo | 13 | 13.00 | 100.00 | 1.00 | 3.00 | 23.08 | 0.12 | 1.00 | 7.69 | 0.04 | 4.00 | 30.77 | 0.17 |
| 33 | Givat-Koach | 13 | 12.00 | 92.31 | 0.72 | 3.00 | 23.08 | 0.12 | 1.00 | 7.69 | 0.04 | 4.00 | 30.77 | 0.17 |
|  |  |  | W195T | | | W207T | | | W259A | | | W263TC | | |
|  |  |  | "+" | "%" | Frequncy | "+" | "%" | Frequncy | "+" | "%" | Frequncy | "+" | "%" | Frequncy |
| 1 | Mt. Hermon | 9 | 4.00 | 44.44 | 0.25 | 6.00 | 66.67 | 0.42 | 8.00 | 88.89 | 0.67 | 8.00 | 88.89 | 0.67 |
| 5 | Qzzrin | 12 | 10.00 | 83.33 | 0.59 | 8.00 | 66.67 | 0.42 | 10.00 | 83.33 | 0.59 | 9.00 | 75.00 | 0.50 |
| 7 | Yehudiyya | 5 | 4.00 | 80.00 |  | 3.00 | 60.00 |  | 4.00 | 80.00 |  | 4.00 | 80.00 |  |
| 8 | Gamla | 12 | 12.00 | 100.00 | 1.00 | 10.00 | 83.33 | 0.59 | 3.00 | 25.00 | 0.13 | 11.00 | 91.67 | 0.71 |
| 9 | Rosh-Pinna | 11 | 9.00 | 81.82 | 0.57 | 8.00 | 72.73 | 0.48 | 10.00 | 90.91 | 0.70 | 9.00 | 81.82 | 0.57 |
| 11 | Tabiha | 22 | 17.00 | 77.27 | 0.52 | 12.00 | 54.55 | 0.33 | 20.00 | 90.91 | 0.70 | 21.00 | 95.45 | 0.79 |
| 16 | Mt. Gilboa | 13 | 7.00 | 53.85 | 0.32 | 7.00 | 53.85 | 0.32 | 13.00 | 100.00 | 1.00 | 11.00 | 84.62 | 0.61 |
| 17 | Mt. Gerizim | 14 | 11.00 | 78.57 | 0.54 | 10.00 | 71.43 | 0.47 | 11.00 | 78.57 | 0.54 | 12.00 | 85.71 | 0.62 |
| 18 | Gitit | 13 | 8.00 | 61.54 | 0.38 | 11.00 | 84.62 | 0.61 | 11.00 | 84.62 | 0.61 | 10.00 | 76.92 | 0.52 |
| 19 | Kokhav Hashahar | 9 | 6.00 | 66.67 | 0.42 | 3.00 | 33.33 | 0.18 | 7.00 | 77.78 | 0.53 | 5.00 | 55.56 | 0.33 |
| 23 | Jaba | 12 | 6.00 | 50.00 | 0.29 | 8.00 | 66.67 | 0.42 | 9.00 | 75.00 | 0.50 | 10.00 | 83.33 | 0.59 |
| 24 | Amirim | 12 | 6.00 | 50.00 | 0.29 | 11.00 | 91.67 | 0.71 | 12.00 | 100.00 | 1.00 | 11.00 | 91.67 | 0.71 |
| 25 | Nahef | 9 | 8.00 | 88.89 | 0.67 | 7.00 | 77.78 | 0.53 | 9.00 | 100.00 | 1.00 | 8.00 | 88.89 | 0.67 |
| 26 | Achihood | 2 | 0.00 | 0.00 |  | 2.00 | 100.00 |  | 2.00 | 100.00 |  | 2.00 | 100.00 |  |
| 28 | Beit-Oren | 16 | 7.00 | 43.75 | 0.25 | 12.00 | 75.00 | 0.50 | 13.00 | 81.25 | 0.57 | 15.00 | 93.75 | 0.75 |
| 29 | Daliyya | 8 | 7.00 | 87.50 | 0.65 | 6.00 | 75.00 | 0.50 | 8.00 | 100.00 | 1.00 | 8.00 | 100.00 | 1.00 |
| 30 | Bat-Shelomo | 13 | 9.00 | 69.23 | 0.45 | 13.00 | 100.00 | 1.00 | 11.00 | 84.62 | 0.72 | 10.00 | 76.92 | 0.52 |
| 33 | Givat-Koach | 13 | 6.00 | 46.15 | 0.27 | 9.00 | 69.23 | 0.45 | 12.00 | 92.31 | 0.72 | 13.00 | 100.00 | 1.00 |
|  |  |  | W263TA | | | W276A | | | W288CG | | | W288AG | | |
|  |  |  | "+" | "%" | Frequncy | "+" | "%" | Frequncy | "+" | "%" | Frequncy | "+" | "%" | Frequncy |
| 1 | Mt. Hermon | 9 | 5.00 | 55.56 | 0.33 | 4.00 | 44.44 | 0.25 | 5.00 | 55.56 | 0.33 | 7.00 | 77.78 | 0.53 |
| 5 | Qzzrin | 12 | 9.00 | 75.00 | 0.50 | 11.00 | 91.67 | 0.71 | 4.00 | 33.33 | 0.18 | 9.00 | 75.00 | 0.50 |
| 7 | Yehudiyya | 5 | 3.00 | 60.00 |  | 5.00 | 100.00 |  | 1.00 | 20.00 |  | 4.00 | 80.00 |  |
| 8 | Gamla | 12 | 6.00 | 50.00 | 0.29 | 9.00 | 75.00 | 0.50 | 5.00 | 41.67 | 0.24 | 10.00 | 83.33 | 0.59 |
| 9 | Rosh-Pinna | 11 | 9.00 | 81.82 | 0.57 | 9.00 | 81.82 | 0.57 | 6.00 | 54.55 | 0.33 | 7.00 | 63.64 | 0.40 |
| 11 | Tabiha | 22 | 16.00 | 72.73 | 0.48 | 21.00 | 95.45 | 0.79 | 7.00 | 31.82 | 0.17 | 17.00 | 77.27 | 0.52 |
| 16 | Mt. Gilboa | 13 | 11.00 | 84.62 | 0.61 | 9.00 | 69.23 | 0.61 | 3.00 | 23.08 | 0.12 | 11.00 | 84.62 | 0.61 |
| 17 | Mt. Gerizim | 14 | 10.00 | 71.43 | 0.47 | 10.00 | 71.43 | 0.47 | 0.00 | 0.00 | 0.00 | 10.00 | 71.43 | 0.47 |
| 18 | Gitit | 13 | 12.00 | 92.31 | 0.72 | 11.00 | 84.62 | 0.61 | 6.00 | 46.15 | 0.27 | 13.00 | 100.00 | 1.00 |
| 19 | Kokhav Hashahar | 9 | 6.00 | 66.67 | 0.42 | 7.00 | 77.78 | 0.53 | 7.00 | 77.78 | 0.53 | 7.00 | 77.78 | 0.53 |
| 23 | Jaba | 12 | 4.00 | 33.33 | 0.18 | 10.00 | 83.33 | 0.59 | 5.00 | 41.67 | 0.24 | 10.00 | 83.33 | 0.59 |
| 24 | Amirim | 12 | 12.00 | 100.00 | 1.00 | 11.00 | 91.67 | 0.71 | 6.00 | 50.00 | 0.29 | 8.00 | 66.67 | 0.42 |
| 25 | Nahef | 9 | 9.00 | 100.00 | 1.00 | 9.00 | 100.00 | 1.00 | 6.00 | 66.67 | 0.42 | 5.00 | 55.56 | 0.33 |
| 26 | Achihood | 2 | 1.00 | 50.00 |  | 2.00 | 100.00 |  | 0.00 | 0.00 |  | 2.00 | 100.00 |  |
| 28 | Beit-Oren | 16 | 16.00 | 100.00 | 1.00 | 11.00 | 68.75 | 0.44 | 5.00 | 31.25 | 0.17 | 15.00 | 93.75 | 0.75 |
| 29 | Daliyya | 8 | 8.00 | 100.00 | 1.00 | 8.00 | 100.00 | 1.00 | 3.00 | 37.50 | 0.21 | 8.00 | 100.00 | 1.00 |
| 30 | Bat-Shelomo | 13 | 11.00 | 84.62 | 0.61 | 8.00 | 61.54 | 0.38 | 1.00 | 7.69 | 0.04 | 9.00 | 69.23 | 0.45 |
| 33 | Givat-Koach | 13 | 11.00 | 84.62 | 0.61 | 11.00 | 84.62 | 0.61 | 1.00 | 7.69 | 0.04 | 13.00 | 100.00 | 1.00 |
