# Supplementary material for: Molecular evolution of dimeric α-amylase inhibitor genes in wild emmer wheat and its ecological association
Source: BMC Evol Biol. 2008 Mar 24;8:91. doi: 10.1186/1471-2148-8-91 (PMC2324104; doi:10.1186/1471-2148-8-91)
Supplement: Additional file 2 — Nei's genetic distance of the inhibitor genes in the 16 populations. This data showed the genetic distances (D) based on the positive fragment of SNP markers among all population pairs. [file 1471-2148-8-91-S2.doc]

Additional file 2. Nei's genetic distance of the inhibitor genes in the 16 populations.

| Population | 1 | 5 | 8 | 9 | 11 | 16 | 17 | 18 | 19 | 23 | 24 | 25 | 28 | 29 | 30 |
| --- | --- | --- | --- | --- | --- | --- | --- | --- | --- | --- | --- | --- | --- | --- | --- |
| 5 | 0.0838 |  |  |  |  |  |  |  |  |  |  |  |  |  |  |
| 8 | 0.1353 | 0.0690 |  |  |  |  |  |  |  |  |  |  |  |  |  |
| 9 | 0.0575 | 0.0168 | 0.0804 |  |  |  |  |  |  |  |  |  |  |  |  |
| 11 | 0.1242 | 0.0520 | 0.1509 | 0.0507 |  |  |  |  |  |  |  |  |  |  |  |
| 16 | 0.0686 | 0.0564 | 0.1695 | 0.0479 | 0.0645 |  |  |  |  |  |  |  |  |  |  |
| 17 | 0.0970 | 0.0697 | 0.0975 | 0.0633 | 0.0733 | 0.0493 |  |  |  |  |  |  |  |  |  |
| 18 | 0.1388 | 0.1089 | 0.1887 | 0.1032 | 0.0739 | 0.0754 | 0.0733 |  |  |  |  |  |  |  |  |
| 19 | 0.1103 | 0.1205 | 0.2386 | 0.1215 | 0.1214 | 0.1504 | 0.1594 | 0.1513 |  |  |  |  |  |  |  |
| 23 | 0.0569 | 0.0440 | 0.1131 | 0.0602 | 0.0615 | 0.0836 | 0.0998 | 0.1035 | 0.1147 |  |  |  |  |  |  |
| 24 | 0.1553 | 0.0955 | 0.2205 | 0.0710 | 0.1029 | 0.0747 | 0.1079 | 0.1136 | 0.1989 | 0.1676 |  |  |  |  |  |
| 25 | 0.1728 | 0.1136 | 0.1993 | 0.0836 | 0.1203 | 0.0676 | 0.1013 | 0.1198 | 0.2473 | 0.1907 | 0.0714 |  |  |  |  |
| 28 | 0.0898 | 0.0765 | 0.1283 | 0.0637 | 0.1283 | 0.0632 | 0.0818 | 0.0934 | 0.2147 | 0.1233 | 0.0639 | 0.1064 |  |  |  |
| 29 | 0.1809 | 0.1361 | 0.2087 | 0.1222 | 0.1165 | 0.0726 | 0.1183 | 0.0830 | 0.2634 | 0.1964 | 0.0923 | 0.0510 | 0.0869 |  |  |
| 30 | 0.0972 | 0.1044 | 0.1316 | 0.0772 | 0.1463 | 0.0749 | 0.0417 | 0.0977 | 0.2238 | 0.1421 | 0.0830 | 0.0998 | 0.0770 | 0.1296 |  |
| 33 | 0.0970 | 0.0864 | 0.1535 | 0.0911 | 0.0879 | 0.0526 | 0.0733 | 0.0729 | 0.1900 | 0.1065 | 0.0978 | 0.1409 | 0.0476 | 0.0613 | 0.1036 |
