# Supplementary material for: Molecular evolution of dimeric α-amylase inhibitor genes in wild emmer wheat and its ecological association
Source: BMC Evol Biol. 2008 Mar 24;8:91. doi: 10.1186/1471-2148-8-91 (PMC2324104; doi:10.1186/1471-2148-8-91)
Supplement: Additional file 3 — Principal components analysis. This data showed the eigenvalues of correlation matrix, eigenvectors and factor coodinates of 16 populations. [file 1471-2148-8-91-S3.doc]

Additional file 3 Principal components analysis

3A. Eigenvalues of correlation matrix

|  | Eigenvalue | % Total | Cumulative | Cumulative |
| --- | --- | --- | --- | --- |
| Factro 1 | 8.120 | 40.599 | 8.120 | 40.599 |
| Factro 2 | 5.732 | 28.660 | 13.852 | 69.259 |
| Factro 3 | 2.731 | 13.653 | 16.582 | 82.912 |
| Factro 4 | 1.179 | 5.894 | 17.761 | 88.805 |
| Factro 5 | 0.648 | 3.242 | 18.409 | 92.047 |

3B. Eigenvectors

|  | Factor 1 | Factor 2 | Factor 3 | Factor 4 |
| --- | --- | --- | --- | --- |
| Ln | 0.054245 | **0.350610** | 0.258255 | 0.213620 |
| Lt | -0.150005 | 0.139920 | 0.375698 | **0.404168** |
| Al | -0.181019 | 0.286215 | -0.256479 | 0.012862 |
| Tm | 0.279403 | -0.208413 | 0.092343 | 0.113186 |
| Ta | 0.294387 | -0.129495 | 0.084185 | 0.298167 |
| Tj | 0.225601 | -0.293454 | 0.036147 | 0.163754 |
| Td | 0.093109 | **0.357214** | 0.124145 | 0.272579 |
| Tdd | 0.279856 | -0.113805 | 0.157939 | -0.197162 |
| Rn | -0.293863 | 0.161702 | -0.017323 | 0.231140 |
| Rd | **-0.313262** | -0.052034 | 0.156468 | 0.142280 |
| Hu-14 | -0.247254 | -0.289121 | -0.007315 | 0.040598 |
| Hu-an | -0.227133 | -0.308624 | -0.014562 | -0.124507 |
| Dw | -0.231379 | -0.228345 | 0.260581 | 0.020137 |
| Sh | 0.064323 | 0.258365 | **-0.428437** | -0.027712 |
| Th | -0.130287 | 0.238850 | 0.360870 | -0.186068 |
| Trd | 0.249183 | -0.122983 | 0.164736 | 0.299473 |
| Ev | **0.296610** | 0.011074 | -0.120366 | 0.104184 |
| Rv | 0.240000 | 0.265524 | 0.009869 | -0.177564 |
| Rr | 0.244046 | 0.124679 | 0.210061 | -0.270217 |
| Rad | 0.009239 | -0.061392 | **-0.420132** | **0.466314** |

3C. Factor Coodinates of 16 populations

|  | Factor 1 | Factor 2 | Factor 3 | Factor 4 |
| --- | --- | --- | --- | --- |
| Mt. Hermon | -5.70900 | 4.64864 | 0.11659 | 1.50695 |
| Qazerin | 0.98152 | 1.59340 | 2.18154 | -0.17298 |
| Gamla | 1.22402 | 2.02431 | 4.08939 | -1.99381 |
| Rosh-Pinna | -0.55842 | 1.32244 | -0.24429 | 0.56324 |
| Tabiha | 3.67412 | -0.54336 | 1.32039 | 2.10969 |
| Mt. Gilboa | 3.47815 | -0.46752 | 0.18315 | 1.00108 |
| Mt. Gerizim | -0.16168 | 1.91491 | -2.04764 | -1.53411 |
| Gitit | 4.72012 | 0.87858 | -1.74272 | 0.37141 |
| Kokhav Hashahar | 2.56990 | 0.36976 | -1.89647 | -0.20624 |
| J’aba | -0.44760 | -0.28318 | -2.41424 | -1.12899 |
| Amirim | -1.40414 | 1.83248 | -0.70077 | 0.16152 |
| Nahef | -1.47633 | 0.33368 | -0.08637 | -0.32898 |
| Beit-Oren | -4.34070 | -3.18410 | 0.06903 | -0.15577 |
| Daliyya | -1.38577 | -3.47393 | 0.39282 | 0.70776 |
| Bat-Shelomo | -2.33304 | -4.14535 | 0.45592 | 0.16649 |
| Givat-Koach | 1.16884 | -2.82076 | 0.32367 | -1.06728 |
