# Supplementary figures and images for: Molecular evolution of dimeric α-amylase inhibitor genes in wild emmer wheat and its ecological association
Source: BMC Evol Biol. 2008 Mar 24;8:91. doi: 10.1186/1471-2148-8-91 (PMC2324104; doi:10.1186/1471-2148-8-91)

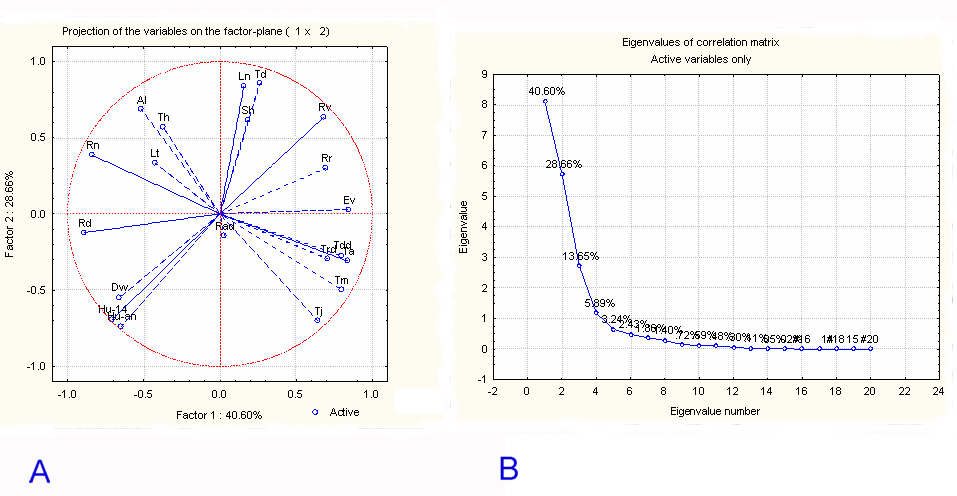

Supplement: Additional file 4 — Principal components analysis of the ecological factors. This data showed the projection of the variables on the ecological factor-plane and eigenvalues of correlation matrix. [file 1471-2148-8-91-S4.bmp]
