# Supplementary material for: Molecular evolution of dimeric α-amylase inhibitor genes in wild emmer wheat and its ecological association
Source: BMC Evol Biol. 2008 Mar 24;8:91. doi: 10.1186/1471-2148-8-91 (PMC2324104; doi:10.1186/1471-2148-8-91)
Supplement: Additional file 5 — Correlations of the Factors. This data showed the correlation of the 20 ecological factors that separated into four groups. [file 1471-2148-8-91-S5.doc]

Additional file 5 Correlations of the Factors

| Group |  | Ln | Lt | Al | Tm | Ta | Tj | Td | Tdd | Rn | Rd | Hu-14 | Hu-an | Dw | Sh | Th | Trd | Ev | Rv | Rr | Rad |
| --- | --- | --- | --- | --- | --- | --- | --- | --- | --- | --- | --- | --- | --- | --- | --- | --- | --- | --- | --- | --- | --- |
| I | **Ln** | 1.00 | 0.60 | 0.32 | -0.18 | 0.02 | -0.40 | 0.90 | -0.03 | 0.20 | -0.11 | -0.68 | -0.77 | -0.40 | 0.25 | 0.64 | 0.00 | 0.06 | 0.61 | 0.38 | -0.31 |
| I | **Lt** | 0.60 | 1.00 | 0.13 | -0.37 | -0.25 | -0.38 | 0.36 | -0.39 | 0.56 | 0.47 | 0.09 | -0.06 | 0.35 | -0.32 | 0.58 | -0.17 | -0.49 | -0.13 | -0.08 | -0.24 |
| I | Al | 0.32 | 0.13 | 1.00 | -0.78 | -0.69 | -0.83 | 0.39 | -0.65 | 0.72 | 0.33 | -0.10 | -0.16 | -0.22 | 0.62 | 0.36 | -0.65 | -0.33 | 0.07 | -0.33 | 0.18 |
| II | **Tm** | -0.18 | -0.37 | -0.78 | 1.00 | 0.93 | 0.94 | -0.14 | 0.75 | -0.84 | -0.57 | -0.19 | -0.18 | -0.18 | -0.25 | -0.53 | 0.74 | 0.57 | 0.23 | 0.41 | 0.02 |
| II | Ta | 0.02 | -0.25 | -0.69 | 0.93 | 1.00 | 0.87 | 0.12 | 0.67 | -0.78 | -0.61 | -0.35 | -0.36 | -0.30 | -0.12 | -0.47 | 0.74 | 0.66 | 0.36 | 0.39 | 0.09 |
| II | Tj | -0.40 | -0.38 | -0.83 | 0.94 | 0.87 | 1.00 | -0.37 | 0.66 | -0.79 | -0.45 | 0.05 | 0.07 | -0.01 | -0.34 | -0.63 | 0.65 | 0.47 | -0.01 | 0.19 | 0.17 |
| II | **Td** | 0.90 | 0.36 | 0.39 | -0.14 | 0.12 | -0.37 | 1.00 | -0.05 | 0.18 | -0.20 | -0.75 | -0.84 | -0.53 | 0.43 | 0.43 | 0.11 | 0.27 | 0.67 | 0.38 | -0.19 |
| II | Tdd | -0.03 | -0.39 | -0.65 | 0.75 | 0.67 | 0.66 | -0.05 | 1.00 | -0.83 | -0.58 | -0.39 | -0.29 | -0.32 | -0.24 | -0.17 | 0.68 | 0.69 | 0.36 | 0.57 | -0.17 |
| III | Rn | 0.20 | 0.56 | 0.72 | -0.84 | -0.78 | -0.79 | 0.18 | -0.83 | 1.00 | 0.76 | 0.33 | 0.24 | 0.34 | 0.10 | 0.46 | -0.56 | -0.59 | -0.41 | -0.47 | 0.06 |
| III | **Rd** | -0.11 | 0.47 | 0.33 | -0.57 | -0.61 | -0.45 | -0.20 | -0.58 | 0.76 | 1.00 | 0.71 | 0.65 | 0.77 | -0.44 | 0.45 | -0.43 | -0.72 | -0.75 | -0.64 | -0.11 |
| III | Hu-14 | -0.68 | 0.09 | -0.10 | -0.19 | -0.35 | 0.05 | -0.75 | -0.39 | 0.33 | 0.71 | 1.00 | 0.96 | 0.85 | -0.53 | -0.18 | -0.31 | -0.62 | -0.91 | -0.73 | 0.08 |
| III | **Hu-an** | -0.77 | -0.06 | -0.16 | -0.18 | -0.36 | 0.07 | -0.84 | -0.29 | 0.24 | 0.65 | 0.96 | 1.00 | 0.82 | -0.56 | -0.15 | -0.28 | -0.54 | -0.89 | -0.63 | 0.04 |
| III | Dw | -0.40 | 0.35 | -0.22 | -0.18 | -0.30 | -0.01 | -0.53 | -0.32 | 0.34 | 0.77 | 0.85 | 0.82 | 1.00 | -0.74 | 0.18 | -0.16 | -0.68 | -0.75 | -0.42 | -0.21 |
| II | **Sh** | 0.25 | -0.32 | 0.62 | -0.25 | -0.12 | -0.34 | 0.43 | -0.24 | 0.10 | -0.44 | -0.53 | -0.56 | -0.74 | 1.00 | -0.15 | -0.25 | 0.30 | 0.50 | 0.05 | 0.31 |
| III | **Th** | 0.64 | 0.58 | 0.36 | -0.53 | -0.47 | -0.63 | 0.43 | -0.17 | 0.46 | 0.45 | -0.18 | -0.15 | 0.18 | -0.15 | 1.00 | -0.33 | -0.37 | 0.14 | 0.16 | -0.52 |
| II | Trd | 0.00 | -0.17 | -0.65 | 0.74 | 0.74 | 0.65 | 0.11 | 0.68 | -0.56 | -0.43 | -0.31 | -0.28 | -0.16 | -0.25 | -0.33 | 1.00 | 0.68 | 0.16 | 0.45 | 0.01 |
| III | **Ev** | 0.06 | -0.49 | -0.33 | 0.57 | 0.66 | 0.47 | 0.27 | 0.69 | -0.59 | -0.72 | -0.62 | -0.54 | -0.68 | 0.30 | -0.37 | 0.68 | 1.00 | 0.47 | 0.46 | 0.18 |
| III | Rv | 0.61 | -0.13 | 0.07 | 0.23 | 0.36 | -0.01 | 0.67 | 0.36 | -0.41 | -0.75 | -0.91 | -0.89 | -0.75 | 0.50 | 0.14 | 0.16 | 0.47 | 1.00 | 0.77 | -0.14 |
| III | **Rr** | 0.38 | -0.08 | -0.33 | 0.41 | 0.39 | 0.19 | 0.38 | 0.57 | -0.47 | -0.64 | -0.73 | -0.63 | -0.42 | 0.05 | 0.16 | 0.45 | 0.46 | 0.77 | 1.00 | -0.29 |
| IV | **Rad** | -0.31 | -0.24 | 0.18 | 0.02 | 0.09 | 0.17 | -0.19 | -0.17 | 0.06 | -0.11 | 0.08 | 0.04 | -0.21 | 0.31 | -0.52 | 0.01 | 0.18 | -0.14 | -0.29 | 1.00 |

* Factor Groups: I Geographical; II Temperature; III Water availability; IV Solar radiation

The factors that were used to do the MR analysis were in bold.
